# Abortion terminology preferences: a cross-sectional survey of people accessing abortion care

**DOI:** 10.1186/s12905-022-02152-8

**Published:** 2023-01-19

**Authors:** Shelly Kaller, Lauren Ralph, Erin Wingo, M. Antonia Biggs

**Affiliations:** 1 Advancing New Standards in Reproductive Health, University of California San Francisco, 1330 Broadway, Suite 1100, Oakland, CA 94612 USA; 2 Present Address: Department of Family and Community Medicine, University of California San Francisco, 995 Potrero Ave, San Francisco, CA 94110 USA

**Keywords:** Abortion, Terminology, Preferences, Stigma, Language, Patient-centered care

## Abstract

**Background:**

Abortion stigma likely affects the terminology abortion patients, providers and the public use or avoid using to refer to abortion care. Knowing the terminology people seeking abortion prefer could help inform the language used in clinical interactions and improve patients’ experiences with abortion care. However, research in the U.S. has not examined patients’ preferences in this area or whether terminology preferences vary by participant characteristics, in the way that experiences of stigma vary across different contexts and communities. This study aims to describe preferred terminology among people presenting for abortion care and to explore the pregnancy-related characteristics associated with these preferences.

**Methods:**

We surveyed abortion patients about their experiences accessing abortion care, including preferred terms for the procedure. Respondents could mark more than one term, suggest their own term, or indicate no preference. We recruited people ages 15–45 seeking abortion from four U.S. abortion facilities located in three states (California, Illinois, and New Mexico) from January to June 2019. We used descriptive statistics and multivariable multinomial logistic regression to explore associations between respondents’ pregnancy-related characteristics and their preferred terminology.

**Results:**

Among the 1092 people approached, 784 (77%) initiated the survey and 697 responded to the terminology preference question. Most participants (57%, n = 400) preferred only one term. Among those participants, “abortion” (43%) was most preferred, followed by “ending a pregnancy” (29%), and “pregnancy termination” (24%). In adjusted multivariable models, participants who worried “very much” that other people might find out about the abortion (29%) were significantly more likely than those who were “not at all” worried (13%) to prefer “ending a pregnancy” over having no preference for a term (adjusted relative risk ratio: 2.68, 95% Confidence Interval: 1.46–4.92).

**Conclusions:**

People seeking abortion have varied preferences for how they want to refer to their abortions, in particular if they anticipate abortion stigma. Findings can be useful for clinicians and researchers so that they can be responsive to people’s preferences during clinical interactions and in the design and conduct of abortion research.

## Background

Abortion is highly stigmatized in the U.S. [1–5]. In June 2022, the U.S. Supreme Court removed federal protections for abortion access, resulting in bans on the procedure in several states, and likely further reinforcing perceptions of stigma [6, 7]. Abortion providers and people who have had abortions often experience abortion stigma and perceive that others look down on them or judge them negatively for performing, having, wanting or seeking an abortion [8, 9]. They may internalize this stigma, feeling shame, guilt, or a need to keep their experience with abortion secret from others, particularly if people fear they may be criminalized for abortion [10]. This widespread and pervasive stigma likely results in abortion patients, providers and the public using varied terms to refer to abortion, including “termination of pregnancy” or “ending a pregnancy”, or intentionally avoiding using the word “abortion”. For example, media portrayals of abortion storylines in pop culture rarely use the term “abortion” [11]. Even politicians who claim to be supportive of abortion rights avoid using the word “abortion” publicly [12], which is likely to perpetuate abortion stigma.

Knowing the terminology people seeking abortion prefer could improve their experiences with abortion care, however, research in the U.S. has not examined patients’ preferences in this area or whether terminology preferences vary by pregnancy-related or sociodemographic characteristics such as age, race, or ethnicity, in the way that experiences of stigma vary across different contexts and communities [3].

Research on people’s preferences for abortion terminology is focused mainly on providers’ preferences, including how language can stigmatize abortion [13–16]. In Scotland, a mixed-methods study with abortion providers found that many preferred using “termination of pregnancy” over “abortion” with patients because it was perceived to be less “harsh” and stigmatizing. However, most of the providers also said that the patients rarely used the term “termination of pregnancy” themselves, more often using “abortion” or “I don’t want the pregnancy.” While its findings may not translate to the U.S., given the politicization of abortion in American culture, one study of over 2000 British women accessing care at abortion facilities found that both the terms “abortion” and “termination of pregnancy” were acceptable and not distressing to patients [17]. Parallel research assessing patients’ preferred terminology to describe their non-viable pregnancy diagnoses found that people preferred “miscarriage”, followed by “early pregnancy loss” and that preferences varied by ethnicity and history of abortion [18]. Patients rated “spontaneous abortion” as the least clear, least heard, and least preferred term. In other settings, including countries in South Asia, notably Bangladesh, as well as West Africa, the use of medication abortion framed as “menstrual regulation” or “missed period pills” before pregnancy is confirmed is less stigmatized and more acceptable than “abortion” [19–21].

Our exploratory study fills a gap in the literature on preferences for abortion terminology by surveying a diverse sample of people presenting for abortion in the U.S. about their preferred terms for their healthcare and identifying pregnancy-related and sociodemographic characteristics associated with those preferences. These findings can be useful for abortion providers to support patient-centered care by better understanding how people conceptualize their abortions and their preferences for how to refer to their abortions.

## Methods

From January to June 2019, we recruited people presenting for abortion while waiting for their appointment at four abortion facilities located in three states (California, Illinois, and New Mexico). A University of California San Francisco (UCSF)*-*trained research assistant approached patients in the waiting rooms to briefly introduce the study and invite patients to screen for eligibility on tablets. Patients were eligible if they were 15 years or older, could speak and read English or Spanish, and were seeking but had not completed their abortion. Patients were ineligible for the study if they were known to be pre-medicated with narcotics for a planned aspiration procedure. Eligible patients who provided informed consent completed a self-administered anonymous survey on paper or an iPad. The survey took an average of 20 min to complete. Participants received a $30 gift card to thank them for their time. UCSF’s Institutional Review Board reviewed and approved the study. Additional details including site and patient recruitment details have been described elsewhere [22]. This sample was powered to develop a scale to measure psychosocial burden accessing abortion care.

After drafting the survey instrument based on a review of the literature [23–25], the research team sought feedback from 11 experts, including four clinicians who provide abortion care, two abortion counselors, one clinic director, three abortion researchers, and one lawyer who supports youth in navigating the judicial bypass process. Based on their feedback, we added, removed, and modified survey items.

Next, we held cognitive interviews with 11 patients from three San Francisco Bay Area abortion facilities, to ensure that the survey language was clear and addressed relevant barriers to accessing abortion care. We also asked patients’ comfort level answering the survey items and how answering the items affected their mood or stress levels to ensure that study participation was not burdensome. We added the survey item on abortion terminology preference after cognitive testing because some patients indicated discomfort with the word “abortion” and a preference for other terms. Also based on this feedback, we included language at the beginning of the survey acknowledging that people use different terms but that we would use the terms “end this pregnancy” or “abortion” to include medication abortion, aspiration or surgical abortion, D&E (dilation and evacuation), D&C (dilation and curettage), or an induced miscarriage or termination.

After we analyzed the study data, we presented findings from the study to the clinic staff at the four abortion facilities that participated in recruitment for the study. We did not collect identifying data from our patient participants so were unable to share the findings directly with them.

### Measures

We assessed our primary outcome, preference on abortion terminology, using the question: “We used the terms ‘end this pregnancy’ and ‘abortion’ to describe what you are seeking today. Given the choice, what word(s) do you prefer to describe it?” Response options included “abortion”, “pregnancy termination”, “ending a pregnancy”, “dilation and curettage (D&C)”, “dilation and evacuation (D&E)”, “no preference”, and an “other” category which allowed participants to write in their response. We coded responses into a 4-part categorical variable which included: (1) abortion, (2) ending a pregnancy, and (3) pregnancy termination for people who selected a single term, and (4) no preference (participants who selected two or more terms or selected “no preference”), which served as our reference category.

To understand whether participants’ circumstances around the pregnancy and abortion may be associated with a preferred term, we included the following independent variables: gestational age (calculated as the difference between the self-reported date of or weeks since last menstrual period and survey date), whether the reason for seeking abortion was because the fetus had a medical condition, pregnancy intention/feelings about the timing of becoming pregnant just before being pregnant, and, as a proxy for anticipated abortion stigma, how worried they were that other people might find out that they ended the pregnancy, which was based on a four-point Likert scale (not at all, a little bit, somewhat, and very much). We chose these independent variables given their documented association with abortion stigma [26]. We also adjusted for the following demographic characteristics: age; race/ethnicity; belonging to a church or religious community; receipt of any governmental financial assistance in the past year (receiving any of the following: Temporary Assistance for Needy Families, WIC, food stamps, social security or disability, another form of government aid); ability to come up with $2,000 in a month if needed; and whether born in the U.S.

In our regression models, we selected reference groups following the guidance of Johfre and Freese [27], using the negation of the variable for binary variables and the lowest quantity group when the variable categorized a quantity. We chose “did not want to become pregnant” as the reference group for pregnancy intention, as other levels of intention “unfold” from this group. Since the meanings of the race/ethnicity categories did not provide a rationale for choosing a reference group, we selected non-Hispanic white because this group had the most frequent responses for not having a preference for a single term.

### Analysis

We ran frequencies on participant characteristics and preference for abortion terminology. We conducted bivariate and fully adjusted multinomial logistic regression analyses estimating relative risk ratios (RRRs). Bivariate analyses, using multinomial regression models, assessed relationships between circumstances around the pregnancy and participant demographic characteristics with abortion terminology preference, including clinic site as a covariate to adjust for clustering of observations by site. The fully adjusted model, using multinomial regression, examined whether circumstances around the pregnancy were associated with preference for a single term, adjusting for participant demographics, pregnancy circumstances, and clinic site. We also conducted a sensitivity analysis to test the robustness of our findings with clinic site included in the fully adjusted model as a random effect rather than a covariate. To handle missing covariate data in our bivariate and adjusted multinomial regression models, we used multiple imputation then deletion using chained equation [28]. We ran ten imputations based on the largest fraction of missing values, including for the outcome variable and covariates [29]. We removed observations with missing outcome data in all regression analyses. We conducted all analyses using Stata 15 (StataCorp, College Station, TX).

## Results

Out of 1092 people we approached about the study, 846 agreed to participate, 824 eligible people initiated the survey, and 784 completed at least one-fifth of the survey. For this analysis, we used the full sample of 784 in our multiple imputation analyses, and then excluded the 87 participants who did not complete the abortion terminology preference survey item which was at the end of the survey, leaving a final analytic sample of 697 participants.

Participant characteristics are shown in Table 1. Most were in their twenties (55%); identified as white, Black/African American, or Hispanic (28%, 28%, and 25%, respectively); did not identify with a church or religious community (73%); and were born in the U.S. (90%). Most were in their first trimester of pregnancy (69%) and either did not want to be pregnant then or at any time (42%) or wanted to be pregnant later or sooner (34%). About half were very much (17%), somewhat (13%), or a little bit worried (19%) that other people might find out they ended the pregnancy. Few (4%) were seeking care because their fetus had a medical condition.

**Table 1:** Distribution of participant characteristics among a sample presenting for abortion (n = 697)

	*n* (%)
Age (years)	
15–19	84 (12.1)
20–24	176 (25.3)
25–29	205 (29.4)
30–34	126 (18.1)
35–45	105 (15.1)
Missing	1 (0.1)
Race/ethnicity	
Non-Hispanic white	197 (28.3)
Hispanic, any race	173 (24.8)
Black, non-Hispanic	195 (28.0)
Asian/Pacific Islander, non Hispanic	45 (6.5)
Mixed race or other race/ethnicity	83 (4.0)
Missing	4 (0.6)
Church or religious community	
None/don't know	507 (72.7)
Protestant/other Christian	115 (16.5)
Catholic	45 (6.5)
Other (Islamic/Jewish/Hindi/Buddhist)	19 (2.7)
Missing	11 (1.6)
Received any government assistance in past year	
No	381 (54.7)
Yes	316 (45.3)
Could come up with $2,000 in a month if needed	
No	492 (70.6)
Yes	191 (27.4)
Missing	14 (2.0)
U.S. born	
No, born in another country	66 (9.5)
Yes, born in United States	628 (90.1)
Missing	3 (0.4)
Gestational age (weeks)	
≤ 12	481 (69.0)
13–19	103 (14.8)
≥ 20	103 (14.8)
Missing	10 (1.4)
Reason for seeking care is fetus has medical condition	
No	670 (96.1)
Yes	27 (3.9)
Pregnancy intention (feelings about becoming pregnant before pregnant)	
Mistimed, wanted to be pregnant later or sooner	237 (34.0)
Wanted to be pregnant then	27 (3.9)
Did not want to be pregnant then or at any time in the future	295 (42.3)
Not sure what wanted	135 (19.4)
Missing	3 (0.4)
Felt worried other people might find out they ended the pregnancy	
Not at all	342 (49.1)
A little bit	135 (19.4)
Somewhat	93 (13.3)
Very much	121 (17.4)
Missing	6 (0.9)
Site	
A	195 (28.0)
B	197 (28.3)
C	207 (29.7)
D	98 (14.1)

When given the option to select their preferred terms, 57% (n = 400) of the 697 selected only one term (Table 2). “Abortion” was the most commonly selected single term, chosen by 43% of participants. “Ending a pregnancy” and “pregnancy termination” were the next most commonly preferred single terms. These three terms were also the most frequently selected, in the same order among the 297 participants who selected multiple terms. Participants (n = 16) who chose only “D&C”, D&E”, and one who suggested an “other” term, writing in “miscarriage”, were excluded from regression analyses due to insufficient sample size.

**Table 2:** Preferred abortion terms among people presenting for abortion care (n = 697)

We used the terms "end this pregnancy" and "abortion" to describe what you are seeking today. Given the choice, what word(s) do you prefer to describe it?	Participants who preferred a single term	Participants who had no preference for a single term
	n (%)	n (%)
Total	400 (57.4)	297 (42.6)
Preferred term^a^		
Abortion	172 (43.0)	125 (42.1)
Ending a pregnancy	117 (29.3)	116 (39.1)
Pregnancy termination	95 (23.8)	106 (35.7)
D&E	9 (2.3)	12 (4.0)
D&C	6 (1.5)	0 (0)
Other	1 (0.3)	3 (1.0)
None, no preference for terms	N/A	169 (56.9)

^a^Participants could select more than one term

In Tables 3 and 4, respectively, we present bivariate and multivariable regression analyses of participants preferring a single term by circumstances related to the pregnancy and abortion and demographic characteristics. Participants who were not sure if they wanted to become pregnant (34%) were significantly more likely than participants who did not want to be pregnant then or in the future (23%) to prefer “abortion” as a term, rather than to have no preference for a term, in bivariate (*p* = 0.002) but not in the fully adjusted models (*p* = 0.052). Participants who reported worrying “very much” about other people finding out about the abortion (29%) were significantly more likely than those who were “not at all” worried (13%) to prefer “ending a pregnancy” over having no preference for a term in the bivariate (*p* = 0.001) and fully adjusted models (adjusted relative risk ratio: 2.68, 1.46–4.92). Demographic covariates including age and race/ethnicity were also significantly associated with a preference for a term. The oldest participants ages 35–45 (compared to the youngest participants ages 15–19) preferred “pregnancy termination”, rather than having no preference in the bivariate and in the fully adjusted analyses. Black participants or participants identifying with more than one race or another race/ethnicity (compared to non-Hispanic white participants) preferred “abortion”, rather than having no preference in the bivariate and in the fully adjusted analyses. There were statistically significant differences in abortion terminology by site in the bivariate, but not fully adjusted models.

**Table 3:** Bivariate associations between participant characteristics and preferred abortion term among people accessing abortion (n = 681)

	Expressed no preference for a single term (reference) n (%)	Preferred "abortion" as only term n (%)	Preferred "ending a pregnancy" as only term n (%)	Preferred "pregnancy termination" as only term n (%)
13+ weeks gestation	93 (47.2)	42 (21.3)	38 (19.3)	24 (12.2)
Reason for seeking care is fetus has medical condition	8 (33.3)	1 (4.2)	8 (33.3)	7 (29.2)
Pregnancy intention (feelings about becoming pregnant before pregnant)				
Mistimed, wanted to be pregnant later or sooner	108 (46.6)	52 (22.4)	37 (16.0)	35 (15.1)
Wanted to be pregnant then	7 (26.9)	7 (26.9)	7 (26.9)	5 (19.2)
Did not want to be pregnant then or at any time in the future (reference)	132 (44.9)	68 (23.1)	53 (18.0)	41 (14.0)
Not sure what wanted	50 (39.7)	43 (34.1)*	20 (15.9)	13 (10.3)
Felt worried other people might find out they ended the pregnancy				
Not at all (reference)	142 (42.3)	105 (31.3)	43 (12.8)	46 (13.7)
A little bit	55 (42.3)	29 (22.3)	26 (20.0)	20 (15.4)
Somewhat	48 (51.6)	19 (20.4)	15 (16.1)	11 (11.8)
Very much	48 (41.4)	19 (16.4)	33 (28.5)**	16 (13.8)
Age (years)				
15–19 (reference)	37 (45.7)	25 (30.9)	12 (14.8)	7 (8.6)
20–24	81 (46.6)	36 (20.7)	29 (16.7)	28 (16.1)
25–29	85 (42.9)	56 (28.3)	33 (16.7)	24 (12.1)
30–34	54 (43.6)	33 (26.6)	21 (16.9)	16 (12.9)
35–45	39 (37.9)	22 (21.4)	22 (21.4)	20 (19.4)*
Race/ethnicity				
Non-Hispanic white (reference)	94 (48.0)	32 (16.3)	41 (20.9)	29 (14.8)
Hispanic, any race	79 (47.3)	30 (18.0)	27 (16.2)	31 (18.6)
Black, non-Hispanic	72 (37.3)	74 (38.3)***	24 (12.4)	23 (11.9)
Asian/Pacific Islander, non-Hispanic	19 (42.2)	9 (21.4)	9 (21.4)	5 (11.9)
Mixed race or other race/ethnicity	29 (36.7)	27 (34.2)**	16 (20.3)	7 (8.9)
Belongs to a church or religious community	77 (44.3)	49 (28.2)	27 (15.5)	21 (12.1)
Received any government assistance in past year	129 (42.0)	86 (28.0)	51 (16.6)	41 (13.4)
Could come up with $2,000 in a month if needed	83 (44.6)	42 (22.6)	33 (17.7)	28 (15.1)
U.S. born	262 (42.7)	161 (26.2)	105 (17.1)	86 (14.0)
Site				
A	78 (40.4)	57 (29.5)	35 (18.1)***	23 (11.9)***
B	88 (45.1)	60 (30.8)	28 (14.4)	19 (9.7)
C	92 (45.8)	32 (15.9)**	33 (16.4)	44 (21.9)
D	39 (42.4)	23 (25.0)	21 (22.8)	9 (9.8)

Table presents row percents, calculated from bivariate crosstabs, excluding n = 16 who only chose “D&C”, D&E” or an “other” term. Missing values imputed using multiple imputation with chained equations

*P* values were derived from simple multinomial regression models estimating Relative Risk Ratio, with reference outcome category as “no preference”, adjusted for “site”

*p < 0.05, **p < 0.01, ***p < 0.001

**Table 4:** Multivariable associations between participant characteristics and preferred abortion term among people accessing abortion (n = 681)

	Preferred "abortion" as only term versus no preference		Preferred "ending a pregnancy" as only term versus no preference		Preferred "pregnancy termination" as only term versus no preference	
	aRRR (95% CI)	*p* value	aRRR (95% CI)	*p* value	aRRR (95% CI)	*p* value
13+ weeks gestation (reference: ≤ 12 weeks)	0.71 (0.41–1.25)	0.236	0.75 (0.40–1.38)	0.353	0.67 (0.35–1.29)	0.229
Sought care because fetus has medical condition	0.29 (0.03–2.46)	0.255	2.55 (0.81–8.07)	0.111	2.72 (0.84–8.74)	0.094
Pregnancy intention (feelings about becoming pregnant before pregnant) (reference: Did not want to be pregnant then or at any time in the future)						
Mistimed, wanted to be pregnant later or sooner	0.98 (0.60–1.59)	0.927	0.81 (0.47–1.40)	0.455	1.37 (0.77–2.43)	0.290
Wanted to be pregnant then	2.17 (0.66–7.16)	0.204	2.16 (0.64–7.32)	0.213	2.24 (0.60–8.37)	0.229
Not sure what wanted	1.71 (0.99–2.93)	0.052	1.01 (0.53–1.91)	0.984	1.01 (0.48–2.10)	0.980
Felt worried other people might find out they ended the pregnancy (reference: Not at all)						
A little bit	0.95 (0.54–1.65)	0.844	1.66 (0.90–3.07)	0.104	1.12 (0.58–2.16)	0.735
Somewhat	0.58 (0.31–1.08)	0.086	1.07 (0.53–2.18)	0.853	0.73 (0.34–1.58)	0.425
Very much	0.75 (0.40–1.41)	0.372	2.68 (1.46–4.92)	0.001	1.08 (0.53–2.19)	0.832
Age (years) (reference: 15–19)						
20–24	0.61 (0.31–1.23)	0.168	1.10 (0.49–2.50)	0.815	1.82 (0.70–4.7)	0.221
25–29	0.87 (0.45–1.71)	0.696	1.13 (0.50–2.54)	0.772	1.61 (0.60–4.28)	0.341
30–34	0.91 (0.44–1.88)	0.791	1.21 (0.51–2.90)	0.666	1.54 (0.54–4.35)	0.417
35–45	0.88 (0.39–1.96)	0.747	1.73 (0.70–4.30)	0.236	3.11 (1.08–8.92)	0.035
Race/ethnicity (reference: Non-Hispanic white)						
Hispanic, any race	1.28 (0.68–2.42)	0.441	0.94 (0.50–1.78)	0.860	1.19 (0.62–2.27)	0.603
Black, non-Hispanic	2.66 (1.50–4.69)	0.001	0.98 (0.51–1.89)	0.959	1.45 (0.71–2.94)	0.306
Asian/Pacific Islander, non-Hispanic	1.63 (0.61–4.36)	0.332	1.36 (0.50–3.72)	0.543	1.19 (0.37–3.88)	0.772
Mixed race or other race/ethnicity	2.82 (1.42–5.62)	0.003	1.45 (0.68–3.08)	0.332	0.74 (0.28–1.95)	0.545
Belongs to a church or religious community	1.00 (0.63–1.59)	0.997	0.88 (0.52–1.51)	0.653	0.71 (0.40–1.28)	0.253
Received any government assistance in past year	1.08 (0.72–1.63)	0.711	0.98 (0.61–1.56)	0.918	0.95 (0.58–1.58)	0.856
Could come up with $2,000 in a month if needed	0.79 (0.49–1.28)	0.340	0.93 (0.55–1.55)	0.773	0.96 (0.55–1.67)	0.875
U.S. born (reference: Foreign-born)	1.63 (0.72–3.68)	0.237	1.20 (0.53–2.70)	0.659	1.38 (0.57–3.31)	0.477
Site (reference: A)						
B	1.19 (0.70–2.03)	0.526	0.63 (0.33–1.19)	0.153	0.75 (0.36–1.57)	0.447
C	0.71 (0.37–1.24)	0.289	0.66 (0.33–1.29)	0.223	1.96 (0.94–4.06)	0.072
D	1.20 (0.53–2.70)	0.663	1.08 (0.45–2.61)	0.862	0.97 (0.34–2.80)	0.961

Analyses exclude n = 16 responses selecting only “D&C”, D&E” or an “other” term. The multinomial regression model also adjusted for “site”, in additional to all covariates presented in the table. Missing values imputed using multiple imputation with chained equations

aRRR, adjusted relative risk ratio

When we conducted the sensitivity analysis to examine the findings with clinic site as a random effect, the results remained similar in direction and magnitude. In addition to the significant differences described above by race/ethnicity, age, and feeling worried about other people finding out about the abortion, people who were “not sure what they wanted” when asked about their pregnancy intention were significantly more likely than those who “did not want to be pregnant then or at any time in the future” to prefer “abortion” over having no preference for a term in the fully adjusted model (adjusted relative risk ratio: 1.85, 1.09–3.13).

## Discussion

Our findings reflect a range of preferences for terminology on abortion among people seeking abortion, with “abortion” being the most commonly preferred term, although as many as 43% did not have a preference for a single term. A person’s circumstances around their pregnancy, in particular how they perceive others would react to their abortion, may affect their preference. Study participants who were very worried about others finding out about the abortion were more likely to choose “ending a pregnancy” as their preferred term than to not have a preference for a term, compared to participants who were not at all worried about other people finding out. We also saw differences in terminology preference by age and race/ethnicity. Rather than having no preference for a single term, older participants preferred “pregnancy termination” more than younger participants. Black/African American, mixed race, “other” race/ethnicities chose “abortion” as their preferred term more often than white participants.

This was an exploratory study of abortion terminology preference from a quantitative survey item with response options provided by the researchers (and an “other” response). These initial data can help inform further efforts to explore terminology preferences in a more comprehensive and nuanced way. Although the language in the survey acknowledged preferences for different terms, the survey item related to terminology preferences was located at the end of the survey and may have been influenced by the language preceding it. Having the survey item at the end of the instrument also gave participants an opportunity to reflect throughout the course of the survey on how the language impacted them. Due to the terminology preference item placement at the end of the survey, 15% of the 824 participants who initiated the survey did not complete the survey, and thus this final item. However, the sample of 697 participants was sufficient to examine our exploratory analysis and largely mirrors people seeking abortion nationally, when compared by age group, race/ethnicity, and nativity [30]. While the study was open to all genders, we were unable to present accurate estimates on the gender diversity of our sample due to a miscoding error. Transgender, non-binary, and gender-expansive people may experience stigmatizing care [31] and have different preferences for terminology. The survey was cross-sectional, completed by participants at the time they presented to care, before having seen the provider or early in their visit. Their comfort with terms may have changed over the course of a visit and may have differed in other geographical contexts where the stigma around abortion or use of certain terms may differ. Our survey likely best assesses the language people would use with health professionals or researchers. They may use different terms when talking with friends or family and may also be influenced by terms they hear from others. Finally, we administered the survey at abortion facilities in 3 states with relatively few legal abortion restrictions, thus the findings may not be generalizable to abortion patients in other parts of the U.S., particularly as abortion is banned in some states, or in other countries. We are also unable to compare the patients who were approached and did not take the survey with those who did participate. We also acknowledge that our study explored terminology preferences among people seeking facility-based abortion care, while people seeking self-managed abortion outside of a formal medical setting may use different terminology and experience abortion stigma differently.

Our study is the first to investigate patients’ preferences for abortion terminology in the U.S., an important topic to explore in this context, especially given the complicated political and social environment around abortion. We found similar results to the study of British women in that “abortion” and “termination of pregnancy” were acceptable to most [17]. However, our study had higher proportions of participants who did not have a preference for a term, and out of those with a preference, “abortion” was more popular than the most popular term, “termination of pregnancy”, found in Britain. Our sample was more racially and ethnically diverse than the British study, and we found differences in terminology preferences by these characteristics. Our study was also the first to assess how circumstances around a person’s pregnancy may affect their preference for abortion terminology.

As found in Britain, our study concluded that people have varied terminology preferences and that healthcare professionals should be sensitive to these preferences when communicating with patients. Efforts to avoid the use of the term “abortion” altogether are unwarranted and could further perpetuate abortion stigma [32]. Consistent with a shared decision-making approach recommended for pregnancy-options and abortion counseling [33, 34], abortion counselors can mirror the language used by the patient during the visit or ask patients’ preferences for terminology before introducing a term or assuming patient comfort or discomfort with certain terms. In a study interviewing patients about their preferences for pregnancy-options counseling, respondents wanted providers to assess their specific circumstances around their pregnancy and tailor their counseling appropriately and sensitively [35]. Researchers may also consider asking preferred terms in the beginning of data collection and using the participants’ terms throughout the survey or interview, either verbally or using piped-in text.

When unable to ask someone’s preference for a term, our findings also show that “abortion” is a generally accepted term. Intentional avoidance of term “abortion” is not consistent with most participants’ preferences, and has the potential to further stigmatize abortion or to cause confusion [32]. In a politicized and stigmatized climate around abortion, providers and researchers may choose to use the term “abortion” as an effort to normalize it and increase comfort both with the term and the procedure [34]. Findings from a recent study emphasize how naming “abortion” in communications can provide important framing to a message and may affect how receptive someone is to it [36]. Health department professionals were more likely to open an email with “abortion” in the message and headers than without the term.

Some of our findings suggest that abortion stigma may impact people’s terminology preference. People who do not want others to find out about their abortion prefer “ending a pregnancy,” which could be viewed as a more benign, neutral term, and as way of distancing oneself from the abortion. Further research may examine whether the preference for the term “pregnancy termination” among older participants is related to generational differences in stigma toward abortion. Racial/ethnic differences in abortion stigma and terminology preferences may be in part tied to notions about stratified reproduction, an effect of structural racism where the births and childbearing of certain groups of people are less valued by society [37]. Black participants identified more with the term “abortion” which could be due to the lower levels of perceived and internalized abortion stigma observed among this population compared to other groups [1, 2, 9]. An alternative explanation for this finding may be that people who are Black are responding to gendered racism by discounting other’s judgment and instead developing a positive identity about their pregnancy decisions, protecting them from the effects of stigma and judgment [38]. These findings point to the need for additional research investigating the link between racism, stigma, and abortion terminology preference. Given the changing legal landscape around abortion in the U.S., including an increased likelihood of surveillance and criminalization of pregnant people [39], it will also be important to explore how abortion stigma and terminology preferences change over time, as fear and secrecy around abortion increase and legal restrictions reinforce stigmatized views that abortion is morally wrong [40].

## Conclusions

This study identifies the need to use abortion terms that are destigmatizing and sensitive to patients and recognizes that people have different preferences for how they want to refer to their abortions. While there was a range of preferences in this study, “abortion” was the most accepted single term. Additional research should be conducted on this topic, including qualitative exploration to further understand how people’s experiences and contexts influence their terminology preferences. These efforts could further investigate how stigma affects terminology preferences, in particular generationally across the lifespan and among those who prefer to keep their abortions private and do not want others to know about their abortions. It is also important to understand more about how race intersects with these preferences, including the relationship between experiences with racism and abortion stigma, so that abortion care and research respect people’s lived experiences without perpetuating stigma.

## Data Availability

The dataset generated and analyzed during the current study are available in the Dryad repository, https://datadryad.org/stash/share/zPZQMcnP2QwwimHw48Od8TUsa1sGwcULJI98g1fOi7U (https://doi.org/10.7272/Q61C1V4P).
